# Effects of cold sensitivity in the extremities on circulating adiponectin levels and metabolic syndrome in women

**DOI:** 10.1186/s12906-017-1658-7

**Published:** 2017-03-09

**Authors:** Ah Yeon Park, Seongwon Cha

**Affiliations:** Mibyeong Research Center, Korea Institute of Oriental Medicine, 1672 Yuseongdae-ro, Yuseong-gu, Daejeon, 34054 Republic of Korea

**Keywords:** Cold hypersensitivity in the hands and feet, High-molecular-weight adiponectin, Metabolic syndrome, Emotional cold stress, Extremities

## Abstract

**Background:**

In adipose tissues, adipokine levels, including adiponectin and leptin, are involved in insulin sensitivity and are reciprocally induced by cold temperature stress. Thermogenic response in the extremities (hands and feet) against cold stress can be negatively related to fat mass accumulation, particularly in the abdomen. However, the relationship between the sensation of cold in the extremities and circulating levels of adipokines is not fully understood. Here, we investigated whether adipokine levels are associated with cold hypersensitivity in the hands and feet (CHHF), independent of body mass, and whether the CHHF is related to metabolic syndrome (MS).

**Methods:**

Associations of the CHHF with serum levels of adipokines and MS risk were evaluated in 1021 Koreans (372 men and 649 women), using a linear regression model while controlling for thermogenic factors and a logistic regression model, respectively.

**Results:**

The adiponectin levels were positively associated with the CHHF, particularly in women, irrespective of thermogenic factors, including body mass index (β = 1.23 μg/mL, 95% confidence interval [1.04–1.45]). Logistic regression analysis for MS risk via the CHHF showed that there was a significant inverse association in women (odds ratio = 0.449, 95% confidence interval [0.273–0.737]).

**Conclusions:**

In summary, our founding indicated that the CHHF could induce increased levels of circulating adiponectin and in turn reduce the MS risk in women. Despite complaints of feeling cold, these women could be at lower risk of cardiovascular disease.

## Background

People exhibit differential cold sensitivity at the same environmental temperature, particularly in the extremities, such as the hands and feet. Cold hypersensitivity in the hands and feet (CHHF), which can decrease quality of life, e.g., functional dyspepsia, is considered an important factor in oriental medicine as a form of cold and heat pattern identification, in which the cold and heat refers to someone’s subjective feeling, as well as objective measures of body temperature in the context of warm or cool environments [1]. Treatments with herbal remedies [particularly Korean red ginseng (KRG)] and acupuncture have been used to attempt to relieve hypersensitivity in the extremities [2, 3].

The thermogenic response to a homeothermic state in the extremities, e.g., cold temperature (or emotional stress) [4], varies according to an individual’s fat mass accumulation; hand temperature in obese individuals tends to be higher than that in normal weight individuals [5]. The skin temperature of the extremities, which is negatively correlated with that of the abdomen, can be affected by the abdominal heat retained under an insulating layer of subcutaneous fat since the core heat may be released through the extremities, in which the skin is not insulated by a substantial amount of fat [5]. In addition, individuals with a higher body mass index (BMI) tend to have warmer hands, as shown in a twin study [6].

Fat accumulation is associated with secretion of adipokines from adipose tissue, including increased leptin secretion and decreased adiponectin secretion [7]. These two adipokines affect thermogenesis during cold stress [8, 9]. That is, the adipose tissue is tightly connected with sympathetic nervous system activity through the response to cold-stress via reduced plasma levels of adiponectin (with no changes in leptin levels) [8, 10], whereas adipose tissue interacts with glucose metabolism during cold stress to facilitate diet-induced thermogenesis and increase circulating adiponectin levels, which can be used in glucose metabolism (accompanied by decreased leptin levels) [9]. Thus, these findings suggest that adiponectin and leptin levels associated with fat accumulation may be related to cold sensation in the extremities. However, the relationship between CHHF and circulating adipokines has not been elucidated.

Therefore, in this study, we hypothesized that adipokines, such as adiponectin and leptin (probably from abdominal fat), may be associated with CHHF because the abdominal fat is inversely related to warm temperature of extremities. Because CHHF is more common in women [6, 11] and is probably affected by body mass, the relationship was also assessed after controlling for sex and BMI. In addition, because the leptin to adiponectin ratio (LAR) and high-molecular-weight (HMW) adiponectin have been suggested to indicate insulin resistance and metabolic syndrome (MS) [12–14], we also examined the association between CHHF and MS.

## Methods

### Participants

A total of 1282 participants (467 men and 815 women) were recruited from 20 oriental medicine clinics by the Korea medicine Data Center (KDC) from 2007 to 2009. All participants provided written informed consent to participate in the study, and the study was approved by the Institutional Review Board of the Korea Institute of Oriental Medicine. Participants with a history of cancer treatment, hypertension medication, diabetes medication, dyslipidemia medication, and/or unknown menopausal status were excluded. MS was defined according to the modified guidelines of the National Cholesterol Education Program Adult Treatment Panel III (NCEP ATP III) [15], which stipulated that at least three of the following five criteria had to be met: (1) abdominal obesity with a waist circumference of 90 cm or more for men and 80 cm or more for women; (2) systolic blood pressure of 130 mmHg or more, diastolic blood pressure (DBP) of 85 mmHg or more, or medication for hypertension; (3) triglycerides (TGs) of 150 mg/dL or more; (4) high-density lipoprotein cholesterol (HDLC) of less than 40 mg/dL for men and less than 50 mg/dL for women; and (5) fasting blood glucose of 110 mg/dL or more or medication for hyperglycemia.

### Classification according to the sensation of cold in the extremities

The sensation of cold in both hands and feet was assessed using a questionnaire that asked the participants to rate the usual temperature of their extremities as warm, neutral, cold, and unknown. The questionnaire for CHHF has previously been used in heritability estimation with twin subjects and in the association with functional dyspepsia [1, 6]. Additionally, the reliability (a correlation coefficient of 0.609 via test-retest) and validity (74.5% agreement and 0.487 kappa value, compared to a professional’s examination) of a seven item questionnaire on cold and heat pattern identification, which includes the CHHF questionnaire, has been assessed [16]. The participants were then grouped by their responses as follows: the non-CHHF group consisted of participants who felt warm in both their hands and feet; the CHHF group consisted of participants who felt cold in both their hands and feet; and the intermediate group consisted of participants who felt neutral in either their hands or feet or in both. The 118 participants who could not be classified into one of these three groups were excluded from the following analyses (final cohort: *n* = 1164, including 420 men and 744 women).

### Anthropometric factors and biochemical analyses

Blood pressure was measured manually at the upper part of left arm after sufficient relaxation using a standard sphygmomanometer. Waist-to-hip ratio (WHR) was defined as the waist circumference divided by the hip circumference. Circumferences of the waist and hip were measured horizontally with a tape measure to the nearest 1 mm at the umbilical level and upper margin of the pubis, respectively. Blood samples were drawn from the participants in the morning after overnight fasting for at least 8 h. Biochemical analyses for glucose, TGs, and HDLC were performed by Seoul Clinical Laboratories (SCL, Seoul, Republic of Korea) based on standardized protocols (ADVIA1800; Siemens, USA). Serum samples were stored at −70 °C until analysis.

### Determination of serum adipokine levels

Serum leptin (ng/mL) was measured by Seoul Clinical Laboratories using a radioimmunoassay method with ^125^I and double antibodies. Of the various isoforms of circulating adiponectin, the HMW form is considered the most clinically relevant [17]. Therefore, the serum concentration of HMW adiponectin (μg/mL) was determined using a Quantikine Human HMW adiponectin/Acrp30 immunoassay kit (R&D Systems, Minneapolis, MN, USA). This kit uses a quantitative sandwich enzyme immunoassay technique to measure total HMW adiponectin concentrations. Each serum sample was analyzed in duplicate (intra-assay coefficient of variation < 10% for all assays), and 62 participants were excluded who had more than a two-fold difference between two repeated measurements (final cohort: *n* = 1102; 396 men and 706 women). In addition, 81 participants with blank data (all leptin) were excluded. A total of 1021 participants were included in the final statistical analyses (372 men and 649 women).

### Statistical analysis

Kruskal-Wallis tests were performed to compare the clinical characteristics between the three cold sensation groups. Linear regression analyses were performed to estimate adiponectin levels (ln-transformed) versus CHHF (referenced by non-CHHF) according to the following adjustment models for confounding variables: Model 1 - adjusted for age and/or sex (in women, menopause status); Model 2 - adjusted for model 1 covariates as well as diastolic blood pressure (DBP) and TGs (ln-transformed); and Model 3 - adjusted for Model 2 covariates as well as BMI. Logistic regression analyses were performed to estimate odds ratios (ORs) for MS and the five MS components versus the CHHF by adjustment for age, sex, and menopausal status. Subgroup analysis was performed for both linear and logistic regression analyses after dividing women by the median value of WHR. Results with a *p* value of less than 0.05 were considered significant. All statistical analyses were performed using R version 3.0.2 software (http://www.r-project.org/).

## Results

### Characteristics of participants based on CHHF

The characteristics of the 1021 participants classified into the non-CHHF, intermediate, and CHHF groups are presented in Table 1. Differences in cardiometabolic and anthropometric traits among the three groups were not similar between men and women. BMI and abdominal traits showed differences in men, whereas all traits except fasting blood glucose levels in women were the highest in the non-CHHF group and the lowest in the CHHF group; the opposite trend was observed for HDLC. These trends were consistent with the inverse correlation between abdominal fat and cold sensitivity in the extremities, as reported previously [5].

**Table 1:** Characteristics of participants in the study

		Cold sensation in hands and feet			
Characteristics	All	non-CHHF	Intermediate	CHHF	*P* value^†^
Men					
N	372	122	173	77	
Age (years)	48.9 (14.9)	49.7 (13.3)	48.8 (15.4)	47.6 (16.3)	0.774
Body mass index (kg/m^2^)	24.1 (3.46)	25.1 (3.08)	24.0 (3.24)	22.8 (4.01)	1.07 × 10^-7^
Waist circumference (cm)	87.3 (9.25)	90.4 (8.29)	87.2 (9.41)	82.8 (8.49)	1.31 × 10^-7^
Waist-to-hip ratio	0.933 (0.0618)	0.950 (0.0518)	0.933 (0.0627)	0.906 (0.0656)	1.31 × 10^-5^
Systolic blood pressure (mmHg)	124 (13.9)	123 (12.6)	123 (12.6)	124 (18.1)	0.992
Diastolic blood pressure (mmHg)	79.8 (10.4)	81.4 (10.3)	79.4 (9.16)	78.1 (12.8)	0.100
Fasting blood glucose (mg/dL)	104 (32.4)	104 (34.1)	104 (31.9)	101 (31.0)	0.254
Triglyceride (mg/dL)	147 (89.2)	151 (75.2)	145 (97.9)	144 (90.0)	0.109
HDL cholesterol (mg/dL)	42.4 (10.5)	41.0 (10.0)	42.6 (10.9)	44.0 (10.1)	0.0727
Women					
N	649	130	229	290	
Age (years)	47.9 (15.8)	52.8 (16.4)	47.7 (16.1)	45.7 (14.7)	1.74 × 10^-4^
Body mass index (kg/m^2^)	23.1 (3.34)	24.5 (3.73)	23.6 (3.24)	22.0 (2.85)	2.07 × 10^-14^
Waist circumference (cm)	82.3 (9.93)	86.5 (10.2)	83.7 (9.39)	79.2 (9.27)	2.62 × 10^-13^
Waist-to-hip ratio	0.890 (0.0698)	0.917 (0.0686)	0.899 (0.0642)	0.870 (0.0693)	3.24 × 10^-10^
Systolic blood pressure (mmHg)	119 (14.9)	123 (14.3)	120 (15.0)	115 (14.3)	1.12 × 10^-8^
Diastolic blood pressure (mmHg)	76.1 (11.0)	79.7 (10.6)	77.2 (11.0)	73.6 (10.6)	2.51 × 10^-8^
Fasting blood glucose (mg/dL)	97.0 (23.7)	102 (34.7)	97.1 (22.9)	94.7 (17.0)	0.133
Triglyceride (mg/dL)	116 (75.3)	142 (91.9)	123 (74.8)	99.0 (62.3)	5.76 × 10^-8^
HDL cholesterol (mg/dL)	49.5 (11.9)	47.2 (12.2)	48.9 (11.3)	50.9 (12.1)	1.39 × 10^-3^

Values are presented as means (standard deviations)

Non-CHHF (cold hypersensitivity in the hands and feet): people feel that both hands and feet are warm

Intermediate: people feel that hands and/or feet are neutral

CHHF: people feel that both hands and feet are cold

^†^ *P* value: Kruskal-Wallis test

### Relationship between circulating adipokine levels and sensitivity to cold

**Fig. 1:**
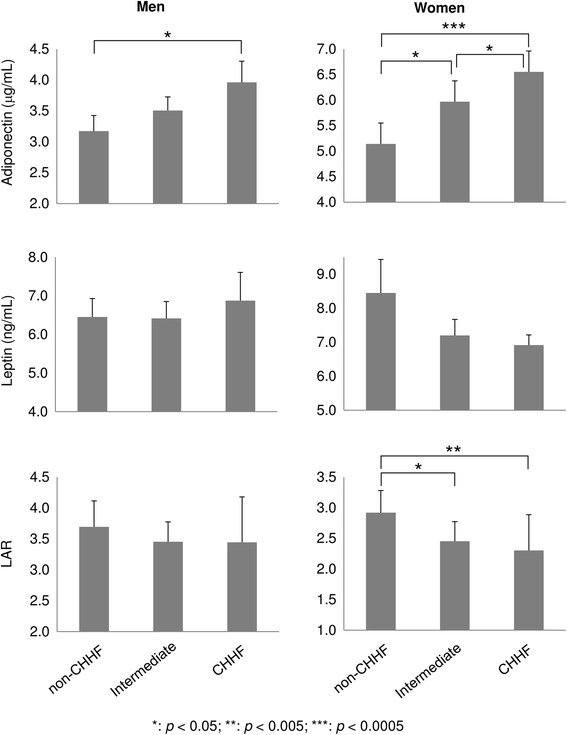
Comparison of adipokine levels according to cold sensitivity in extremities in each group. The *p*-values for different levels of adipokines among cold sensation groups were estimated using Kruskal-Wallis tests (error bars: standard errors). LAR, leptin-to-adiponectin ratio; CHHF, cold hypersensitivity in the hands and feet

From our analysis of the trends in adipokine serum levels, including adiponectin, leptin, and LAR, according to sensitivity to cold in the extremities, women showed significantly increased adiponectin levels and decreased LARs in the order of non-CHHF, intermediate, and CHHF groups, whereas men only exhibited differential levels of adiponectin between the non-CHHF and CHHF groups (Fig. 1). Leptin levels tended to decrease in women, although there were no associations between leptin levels and cold sensation.

A linear regression analysis was performed to determine whether adipokine levels were associated with cold sensitivity after controlling for confounding factors that could influence thermogenesis (i.e., demographic factors [age and/or female menopausal status], vascular function [DBP], lipid fuel [TGs], and/or an anthropometric factor related to the accumulation of fat [BMI]).

The associations of the CHHF (referenced by the non-CHHF) with adiponectin, leptin, and LAR are reported in Table 2. Adiponectin levels were associated with an increased sensitivity to cold, regardless of additional adjustments for DBP, TG, and BMI (Model 1: β = 1.33 μg/mL, *p* = 4.44 × 10^-5^; Model 2: β = 1.23 μg/mL, *p* = 3.28 × 10^-3^; Model 3: β = 1.18 μg/mL, *p* = 2.32 × 10^−2^), although the association was attenuated by the addition of confounding factors. When performing subgroup analysis according to sex, the association for adiponectin levels was maintained and even enriched in women (Model 1: β = 1.40 μg/mL, *p* = 7.87 × 10^-5^; Model 2: β = 1.27 μg/mL, *p* = 3.17 × 10^-3^; Model 3: β = 1.23 μg/mL, *p* = 1.80 × 10^-2^). Additionally, the association between the CHHF and LAR showed a trend similar to that of adiponectin level, except an inverse relationship (β < 0) was observed, and the significant association signal in Model 1 (in all subjects: β = −1.38 μg/mL, *p* = 1.28 × 10^-3^; in women: β = −1.48 μg/mL, *p* = 2.03 × 10^-3^) was reduced in Models 2 and 3 (Table 2). This attenuation could be attributed to the lack of association signal for leptin since there was no synergy from the combination of adiponectin and leptin in the form of a ratio. Overall, the association of cold sensitivity with adiponectin levels was independent of thermogenic factors, including BMI.

**Table 2:** Linear regression analysis of CHHF with adipokine levels

	Model 1		Model 2		Model 3	
Traits^a^	Beta (95% CI)	*P* value	Beta (95% CI)	*P* value	Beta (95% CI)	*P* value
All						
Adiponectin (μg/mL)	1.33 (1.16, 1.53)	4.44 × 10^-5^	1.23 (1.07, 1.40)	3.28 × 10^-3^	1.18 (1.02, 1.35)	2.32 × 10^-2^
Leptin (ng/mL)	−2.32 (−7.67, 1.42)	0.168	−1.49 (−5.06, 2.27)	0.521	1.10 (−3.19, 3.84)	0.59
LAR	−1.38 (−1.68, −1.14)	1.28 × 10^-3^	−1.22 (−1.48, 1.00)	4.62 × 10^-2^	−1.12 (−1.37, 1.09)	0.262
Men						
Adiponectin (μg/mL)	1.26 (−1.02, 1.60)	0.0686	1.21 (−1.04, 1.52)	0.113	1.13 (−1.13, 1.43)	0.332
Leptin (ng/mL)	1.50 (−3.45, 7.80)	0.628	1.64 (−3.23, 8.66)	0.562	1.85 (−3.09, 10.5)	0.163
LAR	−1.27 (−1.77, 1.10)	0.164	−1.21 (−1.67, 1.15)	0.268	−1.09 (−1.53, 1.29)	0.636
Women						
Adiponectin (μg/mL)	1.40 (1.19, 1.66)	7.87 × 10^-5^	1.27 (1.08, 1.50)	3.17 × 10^-3^	1.23 (1.04, 1.45)	1.80 × 10^-2^
Leptin (ng/mL)	−4.38 (−22.2, 1.16)	0.0752	−2.35 (−2.51, 2.22)	0.311	−1.22 (−6.49, 4.39)	0.334
LAR	−1.48 (−1.89, −1.15)	2.03 × 10^-3^	−1.26 (−1.61, 1.01)	0.0611	−1.16 (−1.49, 1.10)	0.233

^a^ln-transformed: adiponectin and LAR

Linear regression, adjusting (Model 1) for age, sex (in all), menopausal status (in women); (Model 2) model 1 covariates, diastolic blood pressure, triglycerides (ln-transformed); (Model 3) model 2 covariates, body mass index

*Abbreviations*: *CHHF* cold hypersensitivity in the hands and feet, *CI* confidence interval, *LAR* leptin-to-adiponectin ratio

Because the correlation between adipokine and cold sensitivity in the extremities could have been affected by differential adiposity among the three groups (non-CHHF, intermediate, and CHHF), the circulating adipokine levels were assessed in terms of abdominal body mass. The high- and low-WHR subgroups corresponded to women with a WHR higher or lower than the median value of WHR for the entire group, respectively. Although no associations were found between CHHF and adiponectin levels in low-WHR women, adiponectin levels were significantly different between the non-CHHF and CHHF groups in high-WHR women after controlling for DBP, TG, BMI, and menopause status (Table 3; β = 1.31 μg/mL, *p* = 2.00 × 10^-2^). These data showed that the levels of adiponectin were associated with cold sensitivity independent of body mass, even in the high-WHR group (Table 2).

**Table 3:** Subgroup analysis for association between CHHF and adipokines after dividing women by the WHR median

	Low WHR^b^		High WHR^b^	
Traits^a^	Beta (95% CI)	*P* value	Beta (95% CI)	*P* value
Adiponectin (μg/mL)	1.11 (−1.16, 1.43)	0.412	1.31 (1.05, 1.65)	2.00 × 10^-2^
Leptin (ng/mL)	1.74 (−11.9, 3.92)	0.571	1.05 (−15.0, 13.6)	0.971
LAR	−1.08 (−1.60, 1.38)	0.709	−1.23 (−1.68, 1.11)	0.191

^a^ln-transformed: adiponectin and LAR

^b^Women (*n* = 649) were divided into two subgroups via the WHR median: low WHR women (*n* = 326; 45 non-CHHF, 105 intermediate, and 176 CHHF) and high WHR women (*n* = 323; 85 non-CHHF, 124 intermediate, and 114 CHHF)

Linear regression, adjusting for age, diastolic blood pressure, triglycerides (ln-transformed), menopausal status, and body mass index

*Abbreviations*: *CHHF* cold hypersensitivity in the hands and feet, *WHR* waist-to-hip ratio, *CI* confidence interval, LAR, Leptin-to-adiponectin ratio

### Relationship between sensitivity to cold and MS

On the basis of the association of HMW adiponectin with MS [14], our results provided evidence for the relationship between CHHF and HMW adiponectin. Therefore, we performed logistic regression analyses of the CHHF for MS and five MS components. The CHHF was associated protectively with MS (OR = 0.465, *p* = 1.01 × 10^-4^) and three components, i.e., low HDLC, high TGs, and large waist circumference (WC) (Table 4). After stratification according to sex, the association of the CHHF with MS was maintained in women, similar to the results for adiponectin (OR = 0.449, *p* = 1.54 × 10^-3^; Table 4). Among the five MS components, high TGs, high BP, and large WC were associated with CHHF in women. Interestingly, CHHF in men was associated with decreased large WC. These associational trends between CHHF and MS risk were consistent with the results presented in Table 1, except HDLC.

**Table 4:** Logistic regression analysis of CHHF with MS and the five components

	All		Men		Women	
Traits	OR (95% CI)	*P* value	OR (95% CI)	*P* value	OR (95% CI)	*P* value
MS	0.465 (0.315, 0.684)	1.01 × 10^-4^	0.690 (0.364, 1.31)	0.256	0.449 (0.273, 0.737)	1.54 × 10^-3^
Low HDLC	0.656 (0.461, 0.932)	1.86× 10^-2^	0.660 (0.367, 1.19)	0.166	0.688 (0.440, 1.07)	0.0994
High TGs	0.467 (0.315, 0.691)	1.40× 10^-4^	0.711 (0.389, 1.30)	0.268	0.422 (0.250, 0.711)	1.21× 10^-3^
High BP	0.712 (0.492, 1.03)	0.0723	1.28 (0.713, 2.31)	0.406	0.578 (0.356, 0.938)	2.65× 10^-2^
High FBG	0.791 (0.499, 1.25)	0.319	0.701 (0.337, 1.46)	0.340	0.957 (0.514, 1.78)	0.888
Large WC	0.298 (0.203, 0.436)	5.35 × 10^-10^	0.300 (0.154, 0.567)	2.12 × 10^-4^	0.343 (0.208, 0.563)	2.38 × 10^-5^

Logistic regression, adjusting for age, sex (in all), and menopausal status (in women)

*Abbreviations*: *CHHF* cold hypersensitivity in the hands and feet, *MS* metabolic syndrome, *OR* odds ratio, *CI* confidence interval, *HDLC*, HDL cholesterol, *TGs* triglycerides; *BP* blood pressure; *FBG*, fasting blood glucose, *WC* waist circumference

The relationship between CHHF and MS was also affected by abdominal body mass, as shown by the WHR subgroup analysis in women. As shown in Table 5, the association between CHHF and MS remained significant in high-WHR women (OR = 0.476, *p* = 1.66 × 10^-2^). Interestingly, the association with large WC was found in low-WHR women but not in high-WHR women. Significant MS risk in high-WHR women was associated with low HDLC and high TGs (low HDLC: OR = 0.498, *p* = 2.91 × 10^-2^; high TGs: OR = 0.357, *p* = 1.93 × 10^-3^).

**Table 5:** Subgroup analysis for the association between CHHF and MS risk after dividing women by WHR median

	Low WHR^a^		High WHR^a^	
Traits	OR (95% CI)	*P*-value	OR (95% CI)	*P*-value
MS	0.669 (0.245, 1.83)	0.435	0.476 (0.259, 0.874)	1.66 × 10^-2^
Low HDLC	1.32 (0.655, 2.66)	0.437	0.498 (0.266, 0.931)	2.91 × 10^-2^
High TGs	0.714 (0.274, 1.86)	0.492	0.357 (0.186, 0.685)	1.93 × 10^-3^
High BP	0.499 (0.218, 1.14)	0.0997	0.717 (0.392, 1.31)	0.281
High FBG	0.835 (0.241, 2.89)	0.777	1.16 (0.563, 2.37)	0.693
Large WC	0.400 (0.188, 0.853)	1.78 × 10^-2^	0.377 (0.142, 1.00)	0.0510

^a^Women (*n* = 649) were divided into two subgroups via the WHR median: low WHR women (*n* = 326; 45 non-CHHF, 105 intermediate, and 176 CHHF) and high WHR women (*n* = 323; 85 non-CHHF, 124 intermediate, and 114 CHHF)

Logistic regression, adjusting age and menopausal status

*Abbreviations*: *MS* metabolic syndrome, *WHR* waist-to-hip ratio, *OR* odds ratio, *CI* confidence interval, *HDLC* HDL cholesterol, *TGs* triglycerides, *BP* blood pressure, *FBG* fasting blood glucose, *WC* waist circumference

## Discussion

In this study, we found that the sensation of cold in the extremities was associated with increased levels of adiponectin (not leptin) and decreased risk of MS in women, independent of body mass. In addition, these trends were enriched in women with high WHR but were absent in women with low WHR. In men, CHHF was only related to abdominal obesity.

Elevation of adiponectin levels by chronic cold exposure enhances the browning of white adipose tissue (WAT) for adaptive thermogenesis [18]. Circulating adiponectin after long-term cold-stress acclimation is involved in glucose metabolism in WAT (and possibly beige adipose tissue), and adaptive thermogenesis is induced by dietary intake rather than by a sympathetic response [9]. Collectively, previous studies have suggested that long-term acclimation to environmental low temperature leads to diet-induced thermogenesis by elevated glucose utilization through the action of adiponectin in WAT. This physiological cold-stress response may resemble the emotional cold-stress response in our study. That is, the increased levels of adiponectin may be induced by cold stress (emotionally mimicked), represented as the CHHF in the context of low environmental temperature. Additionally, because the association between the CHHF and adiponectin levels was observed only in women with a high median WHR, it is possible that the adipose tissue may have an important role in mediating the activity of adiponectin against cold stress. In addition, lower MS risk in CHHF women, even after stratification according to the median WHR, may be affected by increased adiponectin (and possibly by diet-induced thermogenesis) because this adipokine is known to improve insulin sensitivity [9, 19].

However, although women with high abdominal fat tend to have warm hands and feet [5], it is unclear why some high-WHR women show a propensity to for CHHF. A clinical study examining the effects of KRG (a potent vasodilator) on CHHF showed that 8-week treatment with KRG resulted in higher skin temperature in the extremities, lower CHHF severity based on visual analog scale assessment, and less parasympathetic activity from heart rate variability analysis [2]. Accordingly, owing to low sympathetic activity, individuals with CHHF exhibit greater responses in the context of low temperatures and show excessive vasoconstriction in the extremities. A previous study on cold acclimation (8-week exposure) using apolipoprotein E and low-density lipoprotein receptor-knockout mice also supported the concept that sympathetic activity may explain the correlation between adiponectin levels and CHHF [10]. The results of this previous study suggested that normal sympathetic activity can induce uncoupling protein 1-dependent thermogenesis via decreased adiponectin levels, but problems in sympathetic activity can reduce the thermogenic response through maintenance of adiponectin levels. However, in this study, we did not examine sympathetic activity; thus, additional studies are needed to confirm the relationships among CHHF, adiponectin, and sympathetic activity.

CHHF is considered to be a latent Raynaud’s phenomenon (RP) with no color changes [6, 20]. A recent study with the Charleston Heart Study cohort has shown that RP is associated with increased mortality owing to cardiovascular disease (CVD). However, women with CHHF showed lower MS risk (particularly low risk of hypo-HDL-cholesterolemia and hypertriglyceridemia), even in participants with high WHR. Therefore, CHHF may represent very-early-phase RP, although our study participants were selected after excluding individuals with a medication history for CVD. Interestingly, participants who complained of CHHF with no CVD may have healthier blood vessels.

The gender difference in the association between CHHF with adiponectin levels and MS risk could be attributed to estrogen, given that the hormone is involved in the regulation of weight, insulin sensitivity, and body temperature [21–23]. In addition, a previous study has reported that high-level estrogen during the menstrual cycle is associated with less sensitivity to cold stress [24]. Estrogen also improves the peripheral blood circulation by increasing vasodilation [23]. In postmenopausal women, after estrogen levels are notably reduced [25], higher estrogen levels have been associated with higher insulin resistance and lower adiponectin levels [26, 27]. In the present study, high WHR women included a higher proportion of postmenopausal women (64.7%) than low WHR women did (27.0%). Therefore, higher levels of adiponectin in high WHR women may be associated with lower levels of estrogen, which in turn is associated with higher sensitivity of the extremities to cold stress. However, we cannot know the exact relationship between CHHF and adiponectin and estrogen, because we did not measure the estrogen in the studied population.

One of limitations of the current study was that we based our analysis on a questionnaire to determine sensations of cold in the extremities. Therefore, it is difficult to determine whether the changes in adiponectin levels may be caused by emotional cold-stress acclimation, although subjective and objective estimations of finger temperature are similar [28]. Additional studies (with larger sample populations) based on measurements of body temperature and cardiometabolic traits through modulation of the environmental temperature (e.g., seasonal variation) are needed to investigate the link between physiological and emotional cold-stress for adipokine induction, because adipokines such as adiponectin and leptin are associated with changes in cardiometabolic traits [7, 13]. Another limitation was that this was a cross-sectional study, so we cannot know whether the differences in cardiometabolic traits between the three groups (shown in Table 1) induce changes in adiponectin levels or vice versa. To resolve this, it would be necessary to follow the changes in cardiometabolic traits and adipokine levels in a prospective cohort study.

## Conclusions

In conclusion, the results of this study showed that CHHF was correlated with adiponectin levels, independent of body mass, particularly in women with high median WHR. Despite complaints of feeling cold, these women could be at lower risk of MS owing to increased levels of adiponectin and insulin sensitivity.

## Abbreviations

95% CI: 95% confidence interval
BMI: Body mass index
CHHF: Cold hypersensitivity in the hands and feet
CVD: Cardiovascular disease
DBP: Diastolic blood pressure
HDLC: HDL cholesterol
HMW adiponectin: High-molecular-weight adiponectin
KDC: Korea medicine Data Center
KRG: Korean red ginseng
LAR: Leptin-to-adiponectin ratio
MS: Metabolic syndrome
OR: Odds ratio
RP: Raynaud’s phenomenon
TG: Triglyceride
WAT: White adipose tissue
WC: Waist circumference
WHR: Waist-to-hip ratio
